# Oxpentifylline versus placebo in the treatment of erythropoietin-resistant anaemia: a randomized controlled trial

**DOI:** 10.1186/1471-2369-9-8

**Published:** 2008-08-01

**Authors:** David Wayne Johnson, Carmel Mary Hawley, Brenda Rosser, Elaine Beller, Charles Thompson, Robert G Fassett, Paolo Ferrari, Stephen MacDonald, Eugenie Pedagogos, Alan Cass

**Affiliations:** 1 Australian Kidney Trials Network, School of Population Health, University of Queensland, Brisbane, Australia; 2 Department of Nephrology, Princess Alexandra Hospital, Brisbane, Australia; 3 Department of Nephrology, Launceston Hospital, Launceston, Australia; 4 Department of Renal Medicine, Fremantle Hospital, Fremantle, Australia; 5 Department of Nephrology & Transplantation Services, University of Adelaide at the Queen Elizabeth Hospital, Adelaide, Australia; 6 Department of Nephrology, Royal Melbourne Hospital, Melbourne, Australia; 7 The George Institute for International Health, Sydney, Australia

## Abstract

**Background:**

The main hypothesis of this study is that Oxpentifylline administration will effectively treat erythropoietin- or darbepoietin-resistant anaemia in chronic kidney disease patients.

**Methods/design:**

Inclusion criteria are adult patients with stage 4 or 5 chronic kidney disease (including dialysis patients) with significant anaemia (haemoglobin ≤ 110 g/L) for at least 3 months for which there is no clear identifiable cause and that is unresponsive to large doses of either erythropoietin (≥ 200 IU/kg/week) or darbepoetin (≥ 1 μg/kg/week). Patients will be randomized 1:1 to receive either placebo (1 tablet daily) or oxpentifylline (400 mg daily) per os for a period of 4 months. During this 4 month study period, haemoglobin measurements will be performed monthly. The primary outcome measure will be the difference in haemoglobin level between the 2 groups at the end of the 4 month study period, adjusted for baseline values. Secondary outcome measures will include erythropoiesis stimulating agent dosage, Key's index (erythropoiesis stimulating agent dosage divided by haemoglobin concentration), and blood transfusion requirement.

**Discussion:**

This investigator-initiated multicentre study has been designed to provide evidence to help nephrologists and their chronic kidney disease patients determine whether oxpentifylline represents a safe and effective strategy for treating erythropoiesis stimulating agent resistance in chronic kidney disease.

**Trial Registration:**

Australian New Zealand Clinical Trials Registry Number ACTRN12608000199314.

## Background

The development of erythropoiesis stimulatory agents (ESA), such as recombinant human erythropoietin (EPO) and darbepoetin alpha (DPO), has resulted in substantial health benefits for patients with end-stage kidney disease (ESKD), including improved quality of life, reduced blood transfusion requirements, decreased left ventricular mass, diminished sleep disturbance and enhanced exercise capacity [1,2]. Unfortunately, a considerable proportion of such patients exhibit a suboptimal haematologic response to ESA, which in most cases is due to inadequate iron supply to the erythron [3]. Other known causes of ESA-resistance include infection, neoplasia, severe hyperparathyroidism, aluminium intoxication, vitamin B_12 _deficiency, folate deficiency, inadequate dialysis, myelosuppressive agents, haemoglobinopathies, myelodysplasia and antibody-mediated pure red cell aplasia [4]. However, even after exclusion of these conditions, a significant minority (approximately 10%) of patients exhibit ESA-resistant anaemia and have been shown to have greatly increased morbidity and mortality [4]. Inhibition of erythropoiesis by cytokines, such as tumour necrosis factor-α (TNF-α) and interferon-γ (IFN-γ) may play an important role in these patients [5].

Although there is no currently effective treatment for patients with ESA-resistant anaemia, oxpentifylline (pentoxifylline) may represent a promising novel therapeutic strategy. Oxpentifylline has been used for more than twenty years in the treatment of peripheral and cerebral vascular diseases because of its potent haemorrheological properties, which include preservation of erythrocyte water and cation content [6,7]. The drug has subsequently been found to exhibit important anti-inflammatory properties, including anti-apoptotic, anti-oxidant, anti-TNF-α and anti-IFN-γ actions [8-11]. These actions appear to be mediated via inhibition of phosphodiesterase [12]. Two small, prospective, non-randomized studies have demonstrated that oxpentifylline may significantly improve haemoglobin levels in chronic kidney disease patients with ESA-resistant anaemia (vide infra) [13,14] Navarro et al [14] treated 7 anaemic patients with advanced chronic kidney disease (CKD) (creatinine clearance < 30 mL/min) with oxpentifylline (400 mg daily per os) for 6 months. Haemoglobin levels significantly increased from 99 ± 5 to 106 ± 6 g/L (p < 0.01), whilst serum TNF-α concentrations decreased from 623 ± 366 to 562 ± 358 pg/ml (p < 0.01). No changes were observed in untreated controls. Similarly, Cooper and associates [13] administered oxpentifylline (400 mg daily per os) for 4 months to 16 ESKD patients with EPO-resistant anaemia (defined as a haemoglobin level < 107 g/L for 6 months before treatment and an EPO dose ≥ 12,000 IU/week). Among the 12 patients who completed the study, mean haemoglobin concentration increased from 95 ± 9 to 117 ± 10 g/L (p = 0.0001), whilst *ex vivo *T cell generation of TNF-α and IFN-γ were significantly reduced.

Hepcidin may also play a role in ESA-resistant anaemia. This agent is produced by hepatocytes and secreted into the blood in response to iron status, anaemia, hypoxia and pro-inflammatory cytokines, especially IL-6 [15-17] and IL-1 [18]. Recently published data suggests that hepcidin binds to ferroportin (FPN1), a cellular iron exporter, resulting in internalisation and loss of function [19]. FPN1 is highly expressed in macrophages of the reticuloendothelial system and enterocytes in the duodenum and mediates iron release. In macrophages, the binding of hepcidin to FPN1 and its subsequent internalisation would result in iron accumulation within the cell and less release of iron. Patients with CKD may have elevated levels of IL-1, IL-6 and TNF-α [20,21]. It is possible that elevated levels of these cytokines in CKD increase hepcidin production and reduce iron release from macrophages in the bone marrow resulting in reduced availability of iron for erythropoiesis. In fact, prohepcidin was found to accumulate in renal insufficiency [22,23].

In most signaling pathways involving TNF-α and IL-6, IL-6 is a downstream component from TNF via nuclear factor kappa B [24]. Thus, interference with TNF-α production can interfere with IL-6 signalling. In experimental models, oxpentifylline has been shown to reduce IL-6 expression [25-27].

The available studies suggest that oxpentifylline may represent a significant advance in the treatment of ESA-resistant anaemia in chronic kidney disease, but they are limited by their lack of adequate controls and the associated potential for selection, observer and co-intervention biases. A prospective, randomized, double-blind, placebo-controlled trial is required to definitively test the hypothesis that oxpentifylline corrects ESA-resistant anaemia in chronic kidney patients. This study also offers the opportunity to explore the role of the novel peptide, hepcidin, as a possible mediator of iron availability and ESA-resistance.

## Methods/design

Ethics approval for the **H**emoglobin elevation in **E**rythropoietin **R**esistance with **O**xpentifylline (HERO) trial has been obtained from the local Institutional Ethics Committee in all participating centres prior to study initiation and patient enrolment. The study will be performed in accordance with the 2000 Edinburgh, Scotland Revision of the Declaration of Helsinki, the National Health and Medical Research Committee (NHMRC) Statement on Human Experimentation, Joint NHMRC/AVCC Statement and Guidelines on Research Practice, applicable ICH guidelines and the Therapeutic Goods Administration (TGA) – Note for guidance on good clinical practice (CPMP/ICH/135/95) annotated with TGA. Application under the Clinical Trials Notification (CTN) scheme will be required for this protocol.

### Participants

The study population includes adults (18 years or over) with stage 4 or 5 CKD (on dialysis or estimated GFR < 30 ml/min/1.73 m^2^) who are able to give informed consent and who have a haemoglobin concentration < 110 g/L for at least 3 months in spite of EPO dosage ≥ 200 IU/kg/week or DPO dosage ≥ 1 μg/kg/week for at least 1 month. Patients will be recruited by local investigators from participating renal units (outpatients and dialysis units) throughout Australia and New Zealand. The multicentre nature of the study will greatly enhance the generalisability of the trial.

Exclusion criteria include:

1. Patients with a history of psychological illness or condition which interferes with their ability to understand or comply with the requirements of the study.

2. Pregnancy or breast-feeding.

3. Known hypersensitivity to, or intolerance of, oxpentifylline or other methylxanthines, such as caffeine, theophylline or theobromine.

4. Active peptic ulcer disease.

5. Absolute or functional iron deficiency (ferritin < 100 μg/L and/or transferrin saturation < 20%).

6. Vitamin B_12 _or folate deficiency.

7. Parathyroid hormone level > 100 pmol/L.

8. Serum aluminium > 2 μmol/L.

9. Urea reduction ratio < 65% or single pool Kt/V < 1.0 (haemodialysis patients) or total weekly Kt/V < 1.7 (peritoneal dialysis patients).

10. Presence of systemic haematological disease (including antibody-mediated pure red cell aplasia) or known haemoglobinopathy

11. Major surgery, infection, acute myocardial infarction or malignancy within the last 3 months.

12. Melatonin treatment, androgen therapy or blood transfusion within the previous month.

13. Vitamin C therapy at dose greater than 100 mg/day or at a dose which has changed within the last 3 months.

14 Haemorrhagic stroke or severe haemorrhage within the last 3 months.

### Study design

**Figure 1:**
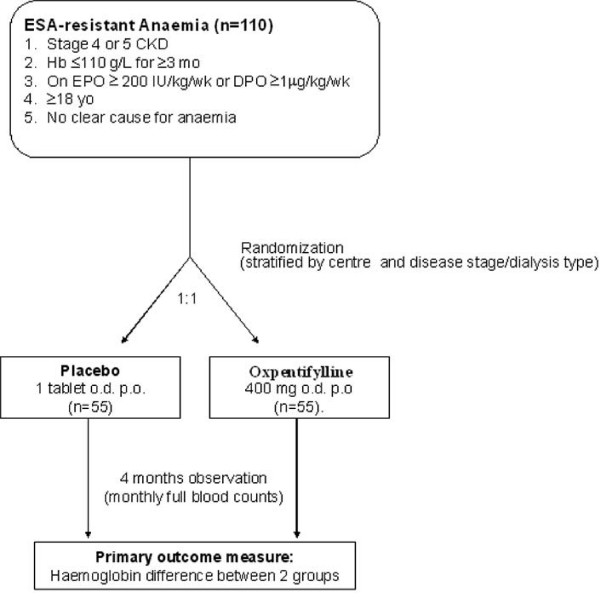
Schema for the HERO Trial.

The study is an investigator-initiated, prospective, double-blind, randomized, placebo-controlled phase 3 trial. Patients will be randomised to one of two treatment groups in equal proportion (Fig. 1). To ensure adequate concealment of allocation, the randomization will be performed using a central computer and web-based link to the central database provided through the Australasian Kidney Trials Network (AKTN). Patients will be randomized in permuted blocks with stratification for centre and disease stage/dialysis type.

### Experimental intervention

The experimental intervention will be oxpentifylline (400 mg daily per os; Trental^®^, Sanofi-Aventis, Sydney, Australia). Oxpentifylline is registered in Australia for treatment of intermittent claudication on the basis of chronic arterial occlusive disease of the limbs. The standard dose is 400 mg three times daily. The dose of oxpentifylline selected for this study (400 mg daily) is lower than the standard dose because the drug may accumulate in renal failure. Moreover, this dose has been shown to be efficacious without significant side effects in 23 ESKD patients studied by Cooper et al [13] and Navarro et al [14]

### Control intervention

The control intervention will be placebo (1 tablet daily per os).

### Concurrent treatments

Iron supplementation will be performed according to usual protocol (note that randomisation will be stratified for centre). Vitamin B and folic acid supplementation are permitted. Melatonin and androgen therapy are proscribed during the study period. If the patient is taking vitamin C, the daily dose will be kept constant throughout the study period. Erythropoietin (EPO) or darbepoetin (DPO) dosages will not be increased during the study, and will only be reduced if haemoglobin levels rise above 125 g/L.

### Blinding

Patients, investigators and outcome assessors will be blinded to the treatment allocation.

### Outcome measures

The primary outcome measure will be the difference in haemoglobin concentration between the oxpentifylline and control groups at the end of the 4 month study period.

Secondary outcome measures include:

a. Difference in the dosage of erythropoiesis stimulating agents (ESA; either erythropoietin or darbepoetin) between the oxpentifylline and control groups at the end of the 4 month study period.

b. Difference in Key's index (ESA dosage divided by haemoglobin concentration) between the oxpentifylline and control groups at the end of the 4 month study period.

c. Difference in frequency of blood transfusion requirements between the oxpentifylline and control groups during the 4 month study period.

d. Occurrence of adverse events in the oxpentifylline and control groups during the 4 month study period.

Exploratory outcome measures that will be studied include the differences in serum concentrations of hepcidin and biomarkers of inflammation (TNF-α, interferon-γ, IL-6 and IL-1) and oxidative stress (isoprostanes, protein carbonyls, plasma catalase and glutathione peroxidase activity) between the oxpentifylline and control groups at the end of the 4 month trial.

### Clinical assessment of outcome

Full blood counts will be measured monthly, as per usual clinical practice. Iron studies will be measured every 2 months. Patients will receive a medical review every 2 months. An individual patient's participation in the study will cease at the end of the 4 month study period. If, before this time, the patient experiences severe anaemia (< 65 g/L), symptomatic anaemia or the patient's attending physician believes that additional therapy is required (eg blood transfusion), they will be considered to have reached an end-point and will be withdrawn from study medication, but will still have blood counts measured monthly and followed for study outcomes.

The number and proportion of subjects who report treatment-emergent adverse events will be summarized for each treatment group. Treatment emergent events include events that start on or after Day 0 of the study [that is the first day of Study Drug administration], and were not present at baseline, or were present at baseline, but increased in severity after the start of the study. The Medical Dictionary for Regulatory Activities [MedDRA] Terminology will be used to classify all adverse events with rESAect to System Organ Class [SOC], high level group term (HLGT), and preferred term.

### Sample size calculations

Prospective power calculations based on unpaired t-test comparisons indicate that the study will have adequate statistical power (90% probability) to detect a clinically significant increase in haemoglobin concentration of 10 g/L, assuming alpha = 0.05 and a population standard deviation of 12 g/L, if 62 patients were recruited in the study (31 in each group). Allowing for a 5% drop-out rate and a 20% non-compliance rate, we aim to recruit a total of 110 patients (55 in each group) across all centres. We anticipate that this will require a recruitment period of 6 months.

### Statistical analyses

Differences between the intervention and control groups with respect to the primary outcome measure (haemoglobin level at 4 months) will be measured by comparison of the mean haemoglobin in each group, adjusted for baseline values (analysis of covariance). Data will be analysed on an intention to treat basis. For patients withdrawing before the end of the 4 month study, their haemoglobin at the time of withdrawal will be taken as their final haemoglobin. A sub-analysis will be performed to determine whether patients with significantly elevated C-reactive protein (CRP) levels (above the local laboratory's reference range) are more likely to have oxpentifylline-responsive anaemia. The data will be analysed by t-test and by repeated measures analysis of covariance. Because of power considerations and the relatively short duration of the study, no interim analysis is planned.

Secondary analyses will be performed by repeated measures analysis of covariance with and without adjustment for baseline characteristics. The analyses used for secondary outcome measures will include unpaired t-test (Key's index), Mann-Whitney U-test (ESA dosage) and chi square test (blood transfusion requirement, adverse drug reactions).

Categorical baseline characteristics (e.g. sex, race, comorbid illnesses, etc) will be summarized with the number and percent of subjects in each treatment group with the characteristic. Quantitative characteristics (e.g. age and weight) will be summarized by mean and standard deviation or median [interquartile range], depending on data distribution. The number and percent of subjects who are randomized, treated with randomized Study Drug, require intervention, prematurely discontinue, and complete the study will be summarized. Fisher's exact test or the Chi-square test will be used to assess treatment group differences in the proportions of subjects who require intervention and who complete the study. The number and percent of subjects will be summarized for each reason for premature discontinuation.

## Discussion

This invesigator-initiated, multicentre Australian and New Zealand study has been designed to provide evidence to help nephrologists and their CKD patients better determine whether Oxpentifylline (Trental^®^) administration will safely and effectively treat erythropoietin- or darbepoietin-resistant anaemia. Given that numerous studies have demonstrated that ESA hyporesponsive patients are at significantly increased risk of mortality [28], novel treatments for correcting anaemia in this group (such as with oxpentifylline) may represent an important strategy for improving clinical outcomes. The multicentre nature of the trial will greatly enhance its generalisability. Moreover, the trial sample size has been carefully and prospectively calculated using a minimum clinically important difference in haemoglobin level of 10 g/L and realistic estimates of trial drop-out and non-compliance rates to minimise the risk of a type 2 statistical error. Demonstration of a significant improvement in haemoglobin levels with oxpentifylline therapy will provide clinicians with an important new strategy for effectively treating ESA-resistant anaemia. On the other hand, a negative study will dissuade clinicians from unnecessarily exposing patients to oxpentifylline (which has a small potential for side effects) if it is proven to lack efficacy with respect to treating ESA-resistant anaemia.

## Abbreviations

AKTN: Australasian Kidney Trials Network; CKD: Chronic kidney disease; CRP: C-reactive protein; CTN: Clinical trials notification; DPO: Darbepoetin; EPO: Erythropoietin; ESA: Erythropoiesis stimulating agent; ESKD: End-stage kidney disease; FPN: Ferroportin; HERO: hemoglobin elevation in erythropoietin resistance with oxpentifylline; IFN-γ: Interferon-gamma; IL-1: Interleukin-1; IL-6: Interleukin-6; IVRS: Interactive voice response system; MedDRA: Medical dictionary for regulatory activities; SOC: System organ class; TGA: Therapeutic Goods Administration; TNF-α: Tumour necrosis factor alpha.

## Competing interests

Financial Competing Interests

The study is funded by grants from the Roche Foundation for Anaemia Research (RoFAR), Amgen Australia and Janssen-Cilag.

Professor Johnson has received consultancy fees and speaker's honoraria from Sanofi-Aventis (manufacturer of oxpentifylline). He has also received consultancy fees, speaker's honoraria, research grants and conference travel sponsorships from Amgen, Janssen-Cilag and Roche (all manufacturers of erythropoiesis stimulating agents).

Dr. Hawley has received conference travel sponsorships from Amgen and Janssen- Cilag.

Professor Ferrari has received speaker's honoraria and conference travel sponsorships from Amgen, Roche and Janssen-Cilag.

Dr McDonald has received speaker's honoraria, and conference travel sponsorships from Amgen and Janssen-Cilag (manufacturers of erythropoiesis stimulating agents).

Associate Professor Alan Cass has received speaker's honoraria and research grants from Amgen, Janssen-Cilag and Roche.

The remaining authors declare that they have no financial competing interests.

Non-Financial Competing Interests

The authors declare that they have no non-financial competing interests.

## Authors' contributions

DJ: Principal Investigator; conceived study; participated in design and co-ordination; helped to draft manuscript; read and approved the final manuscript. CH: Trial Management Committee member; participated in design and co-ordination; helped to draft manuscript; read and approved the final manuscript. BR: Trial Management Committee member; participated in design and co-ordination; helped to draft manuscript; read and approved the final manuscript. EB: Trial Management Committee member; participated in design and co-ordination; provided statistical advice; helped to draft manuscript; read and approved the final manuscript. CT: Trial Management Committee member; participated in design and co-ordination; provided statistical advice; helped to draft manuscript; read and approved the final manuscript. RB: Trial Management Committee member; participated in design and co-ordination; helped to draft manuscript; read and approved the final manuscript. PF: Trial Management Committee member; conceived hepcidin sub-study; participated in design and co-ordination; helped to draft manuscript; read and approved the final manuscript. SM: Trial Management Committee member; participated in design and co-ordination; helped to draft manuscript; read and approved the final manuscript. EP: Trial Management Committee member; participated in design and co-ordination; helped to draft manuscript; read and approved the final manuscript. AC: Trial Management Committee member; participated in design and co-ordination; helped to draft manuscript; read and approved the final manuscript.

## Pre-publication history

The pre-publication history for this paper can be accessed here:


